# Supplementary material for: Selection, biophysical and structural analysis of synthetic nanobodies that effectively neutralize SARS-CoV-2
Source: Nat Commun. 2020 Nov 4;11:5588. doi: 10.1038/s41467-020-19204-y (PMC7642358; doi:10.1038/s41467-020-19204-y)
Supplement: Supplementary file 1 — Supplementary Information [file 41467_2020_19204_MOESM1_ESM.pdf]

SUPPLEMENTARY MATERIAL

Selection, biophysical and structural analysis of synthetic nanobodies that effectively neutralize SARS-CoV-2

Tânia F. Custódio[1], Hrishikesh Das[2], Daniel J Sheward[3,4], Leo Hanke[3], Samuel Pazicky[1], Joanna Pieprzyk[1], Michèle Sorgenfrei[5], Martin A. Schroer[6], Andrey Yu. Gruzinov[6], Cy M. Jeffries[6], Melissa A. Graewert[6], Dmitri I. Svergun[6], Nikolay Dobrev[7], Kim Remans[7], Markus A. Seeger[5], Gerald M McInerney[3], Ben Murrell[3*], B. Martin Hällberg[2,8*] and Christian Löw[1*]

[1] Centre for Structural Systems Biology (CSSB), DESY and European Molecular Biology Laboratory Hamburg, Notkestrasse 85, D-22607 Hamburg, Germany.

[2] Centre for Structural Systems Biology (CSSB) and Karolinska Institutet VR-RÅC, Notkestrasse 85, D-22607 Hamburg, Germany.

[3] Department of Microbiology, Tumor and Cell Biology, Karolinska Institutet, Stockholm 17177, Sweden.

[4] Division of Virology, Institute of Infectious Diseases and Molecular Medicine, Faculty of Health Sciences, University of Cape Town, South Africa.

[5] Institute of Medical Microbiology, University of Zurich, Switzerland.

[6] European Molecular Biology Laboratory (EMBL), Hamburg Outstation c/o Deutsches Elektronen Synchrotron (DESY), Notkestrasse 85, D-22607 Hamburg, Germany.

[7] European Molecular Biology Laboratory (EMBL) Heidelberg, Protein Expression and Purification Core Facility, 69117 Heidelberg, Germany.

[8] Department of Cell and Molecular Biology, Karolinska Institutet, 17177 Stockholm, Sweden.

Supplementary Table 1 – Summary of affinity measurements and neutralization assays

	K_D (nM)	IC_{50} (µg/ml)
Sb12	24.2 ± 4.5	3.7
Sb23	10.6 ± 2.0	0.6
Sb23-Fc	0.22 ± 0.0001	0.007
Sb42	5.0 ± 0.9	1.1
Sb42-Fc	0.19 ± 0.00002	0.07
Sb76	58.1 ± 11.4	9.05
Sb95	43.9 ± 11.9	ND
Sb100	38.7 ± 5.6	2.4
Sb23/12	0.53± 0.0004	0.008

Supplementary Table 2 – Sequence of primers used in this study

Primers for PCR amplification of pCMVExt-Fc plasmid	Fw gacaaaactcacacatgcc<br>Rv ggatccctgaaaatacaggttt
SLIC cloning Sb23-Fc or Sb42-Fc: Primers to amplify Sb23 or Sb42	Fw gaaaacctgtattttcagggatcccaggttcagctggttgag<br>Rv cggtgggcatgtgtgagttttgtcgctcacagtcacttgggt
SLIC cloning Sb23/Sb12: Primers to amplify Sb12 and introduce a GS linker	Fw_1 ggcggtagcggcggaggaggcagcggaggacaggttcagctggttgag<br>Rv_1 gctcacagtcacttgggtac<br>Fw_2 caaggtacccaagtgactgtgagcggaggaggcggtagcggcgga<br>Rv_2 cctcttctgagatgagtttttgttcgccagctcttcctgcgctcacagtcacttgggt
Mutagenic primers to introduce a HindIII restriction site on pSBinit plasmid containing Sb23	Fw tgactgtgagcgcaggaaaagcttgcgaacaaaaactcat<br>Rv atgagtttttgttcgcaagcttttcctgcgctcacagtca

Supplementary Fig. 1

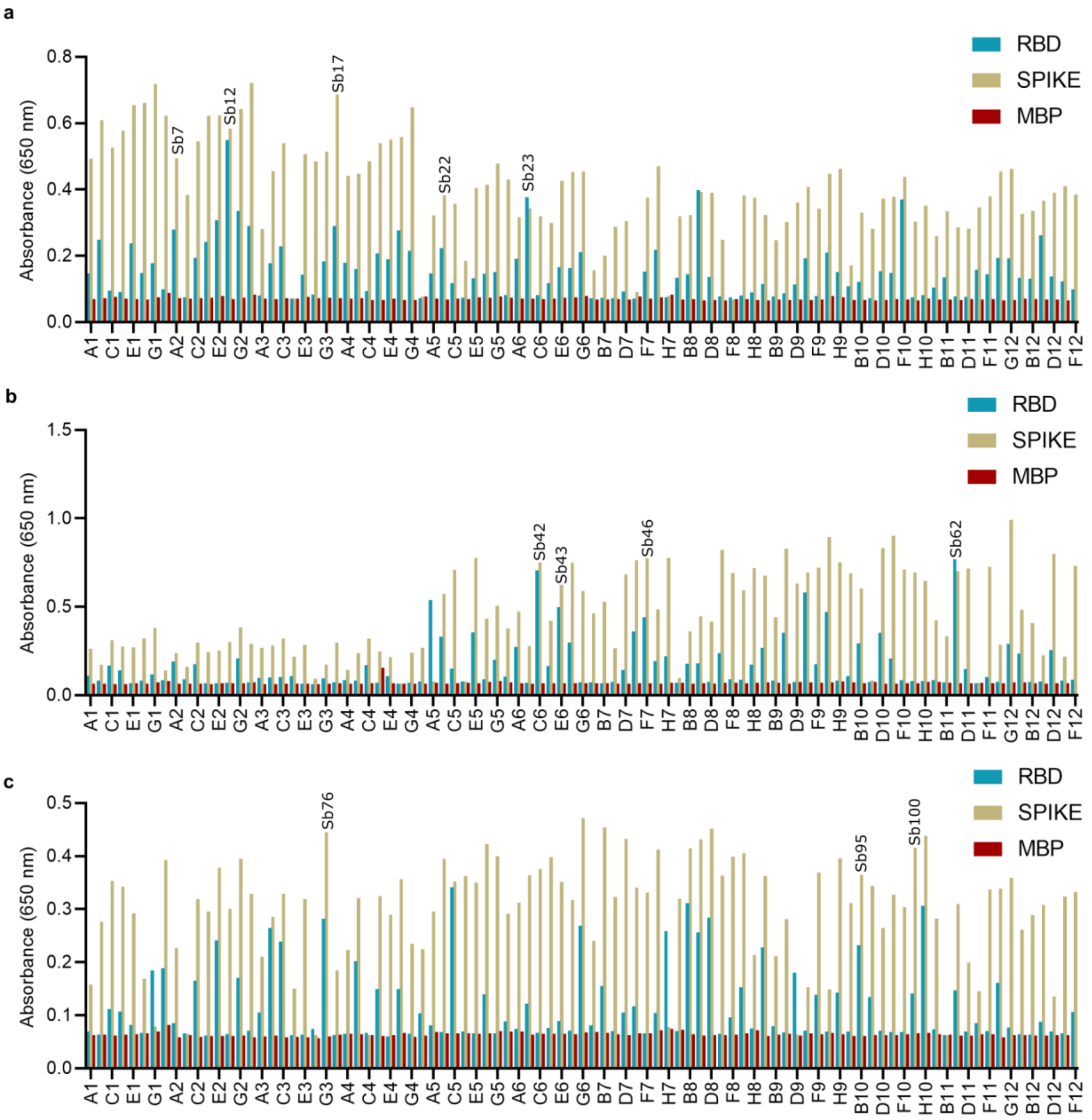

RBD-specific sybodies were determined by ELISA.

94 individual colonies from each of the three libraries, concave **(a)**, loop **(b)** and convex **(c)** were randomly selected and screened against the two target proteins, RBD and spike. The same procedure was performed for the MBP, used as background control signal. RBD or spike signals with ratios above 1.5 compared to the MBP signal, were considered as hits. The corresponding ELISA signals are labelled according to the sybodies that were illustrated or characterized in this work.

Supplementary Fig. 2

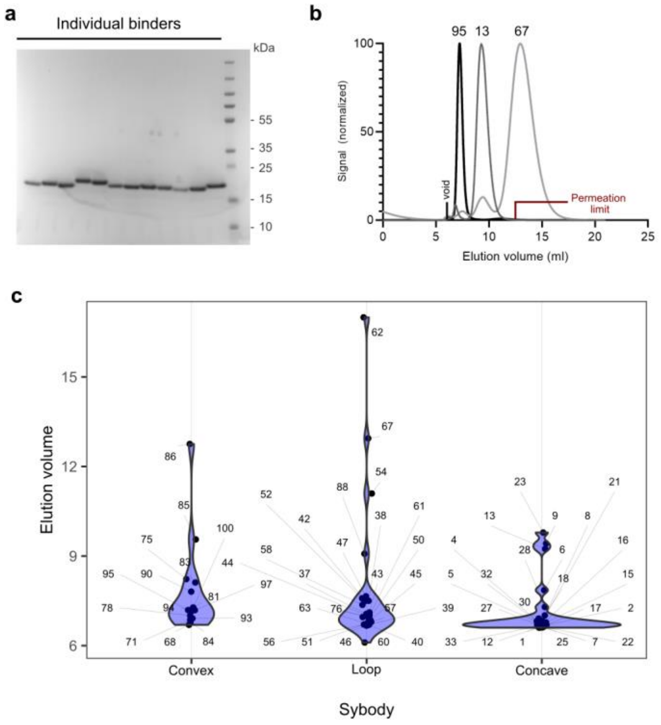

Expression and Purification of 62 unique sybodies.

From 85 unique binders, 62 were expressed and purified. The purified sybodies were analysed by SDS-PAGE **(a)** and gel filtration using a SRT SEC-100 column **(b)**. Sybodies 12, 23, 42, 76, 95, and 100 were expressed and analysed at least two independent times. All other sybodies were only expressed and analysed once. Very few sybodies exhibit column interaction (sticky binders), eluting at or after the permeation limit of columns. **c** Elution volume of all purified binders, grouped by library.

Supplementary Fig. 3

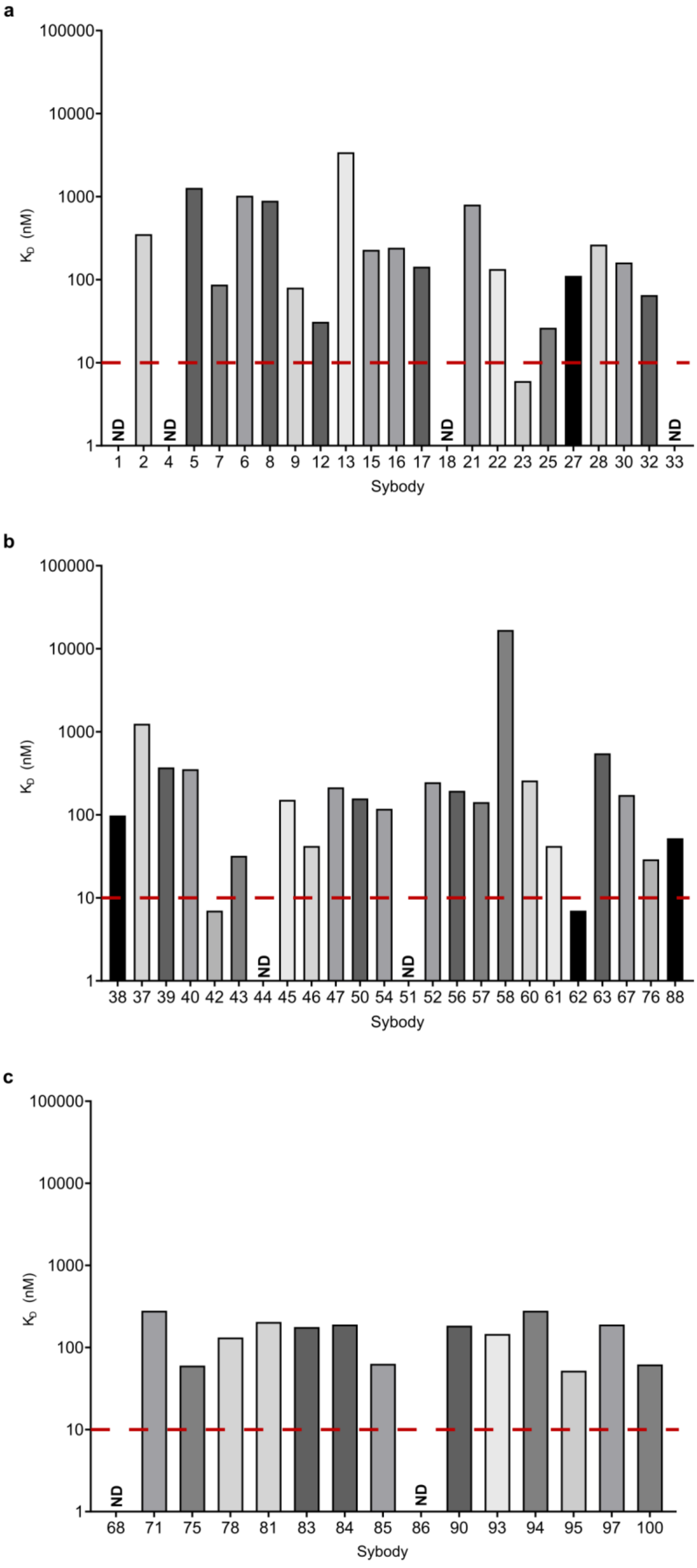

Affinity screening of 62 sybodies.

BLI sensorgrams of immobilized SARS-CoV-2 RBD with individual sybodies were recorded at one concentration (500 nM). The binding curves were fitted to a 1:1 binding model and K_D values were estimated and plotted according to the different libraries concave **(a)**, loop **(b)** and convex (**c**). ND, not determined. The most promising binders, further characterized in this study, are highlighted.

Supplementary Fig. 4

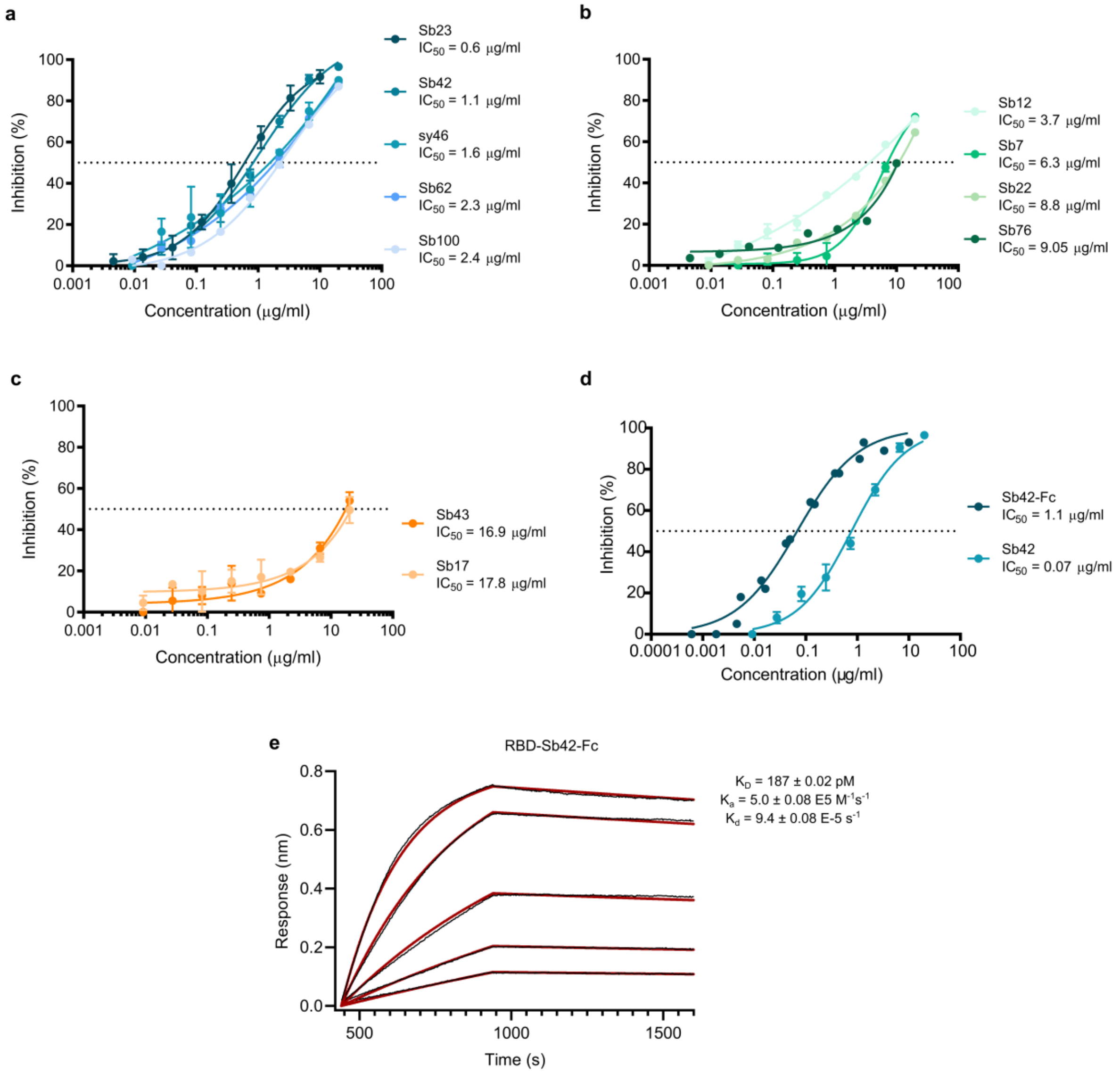

Neutralization capacity of selected sybodies.

SARS-CoV-2 pseudoviruses were incubated with a dilution series of target sybody and grouped according to sybodies that showed the lowest IC_{50} values **(a)**, sybodies that showed IC_{50} values above 3 µg/ml **(b)** or sybodies that showed IC_{50} values above 10 µg/ml (**c**). **d** Neutralization potential of Sb42 is increased when fused to an antibody-derived Fc domain. Data for all assays are mean ± SD of two replicate experiments. **e** BLI sensorgrams of immobilized SARS-CoV-2 RBD with 2-fold serial dilution of 20 nM Sb42-Fc.

Supplementary Fig. 5

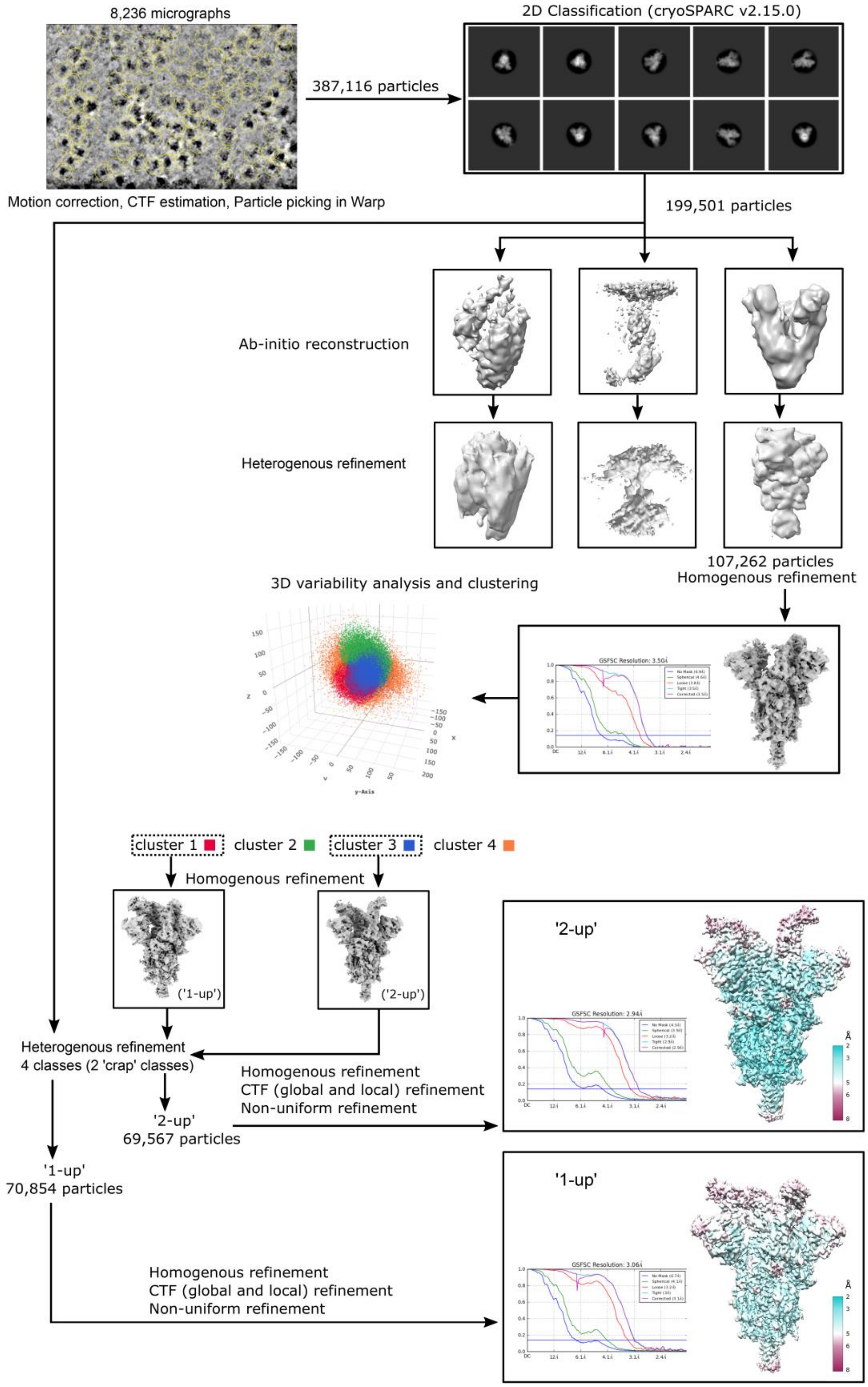

Overview: EM processing performed for the '1-up' and '2-up' reconstructions.

For clarity, the representative micrograph is denoised using Warp [1].

Supplementary Fig. 6

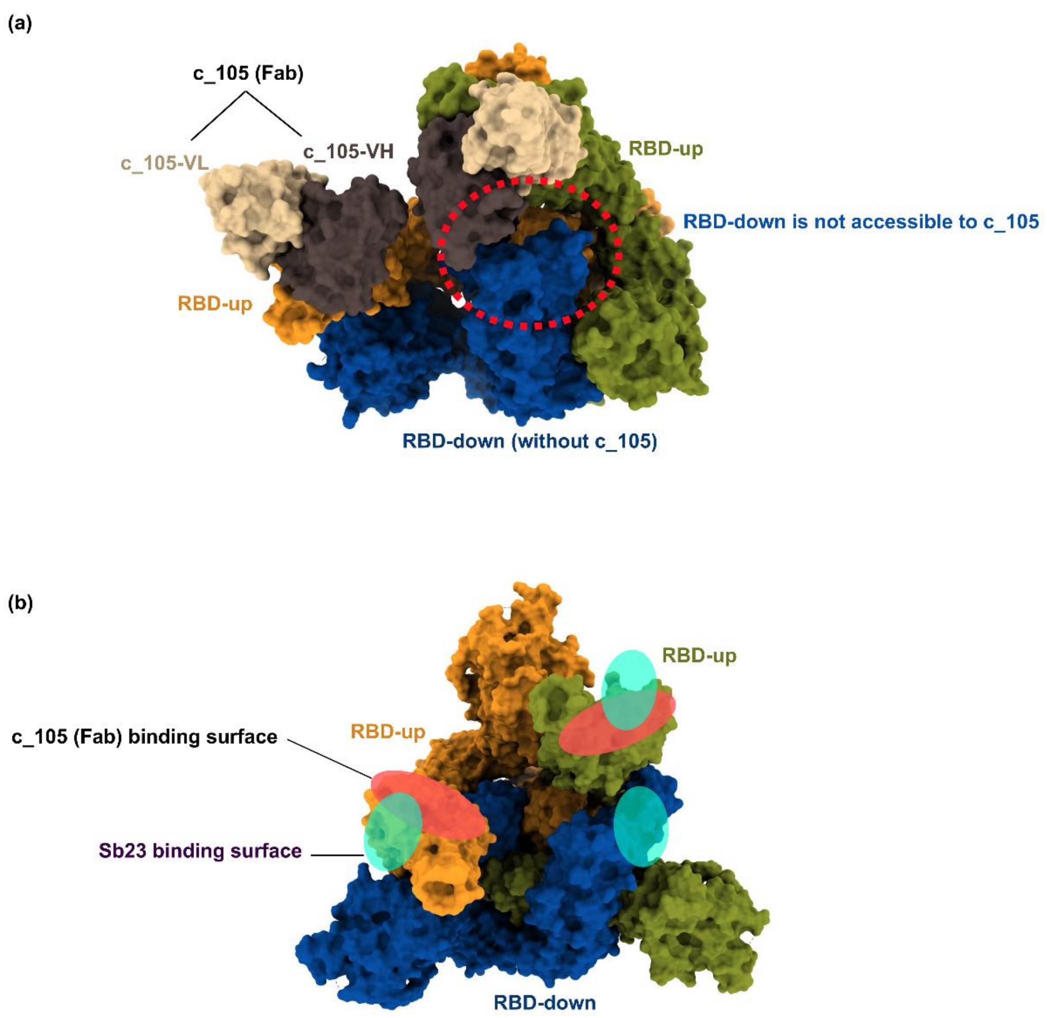

Comparison of Sb23 and FAB_C105 binding.

(a) Cryo-EM reconstruction of FAB_C105 [2] that shows that binding of the RBD in the down state is sterically occluded. (b) top view of SARS-CoV-2 spike showing and comparing the epitopes of Sb23 and FAB_C105.

Supplementary Fig. 7

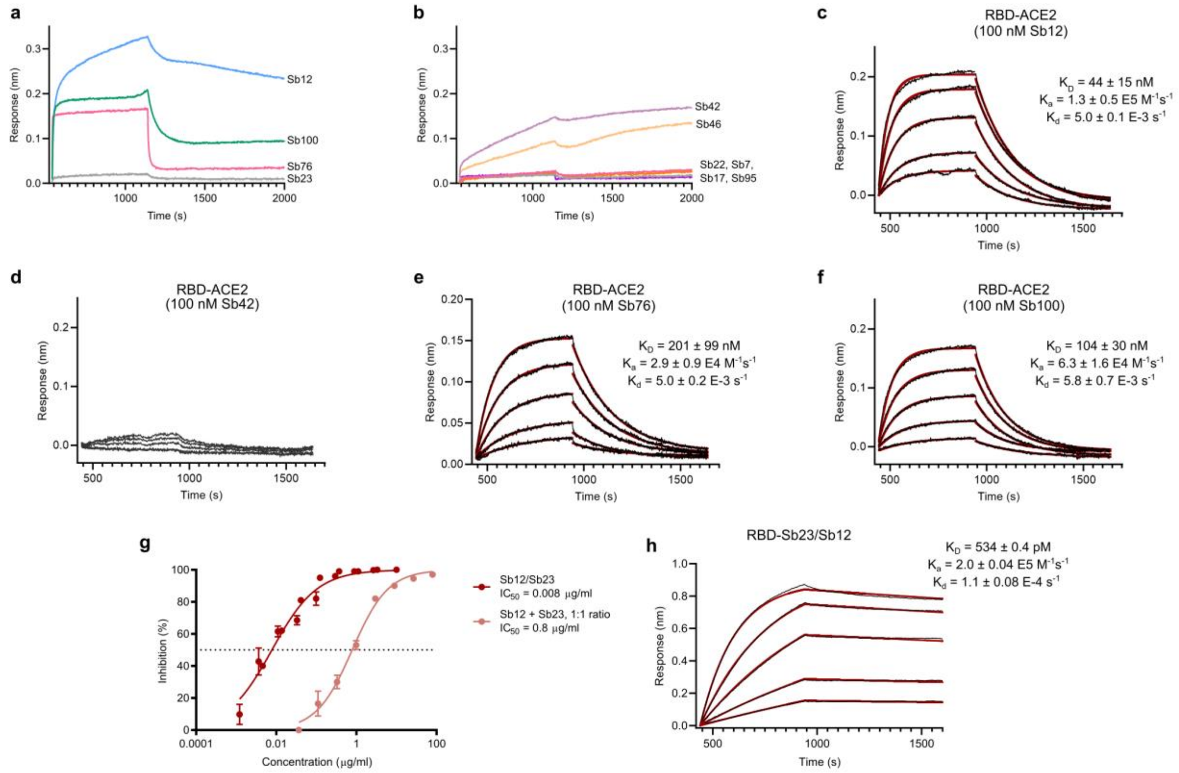

Selected sybodies recognize two distinct epitopes on RBD

BLI sensorgrams of immobilized SARS-CoV-2 RBD in the presence of 100 nM Sb23 with 750 nM of the indicated sybody. Sb12, 76 and 100 recognize a different epitope on RBD than Sb23 **(a)** while the other screened sybodies appear to have the same or overlapping epitopes **(b)**. BLI sensorgrams of immobilized SARS-CoV-2 RBD with ACE2 in the presence 100 nM Sb12 **(c)**, or 100 nM Sb42 **(d)**, or 100 nM Sb76 **(e)**, or 100 nM Sb100 **(f)**. The assay was performed in a concentration range of 200-12.5 nM ACE2 and fit of the data to a 1:1 binding model is shown in red. **g** SARS-CoV-2 spike pseudotyped lentivirus was incubated with a dilution series of Sb23/Sb12 or Sb23 plus Sb12 added in 1:1 molar ratio. The assay was repeated at least in duplicates and the error bars represent the standard deviation. **h** BLI sensorgrams of immobilized SARS-CoV-2 RBD with 2-fold serial dilution of 30 nM Sb23/Sb12.

Supplementary Fig. 8

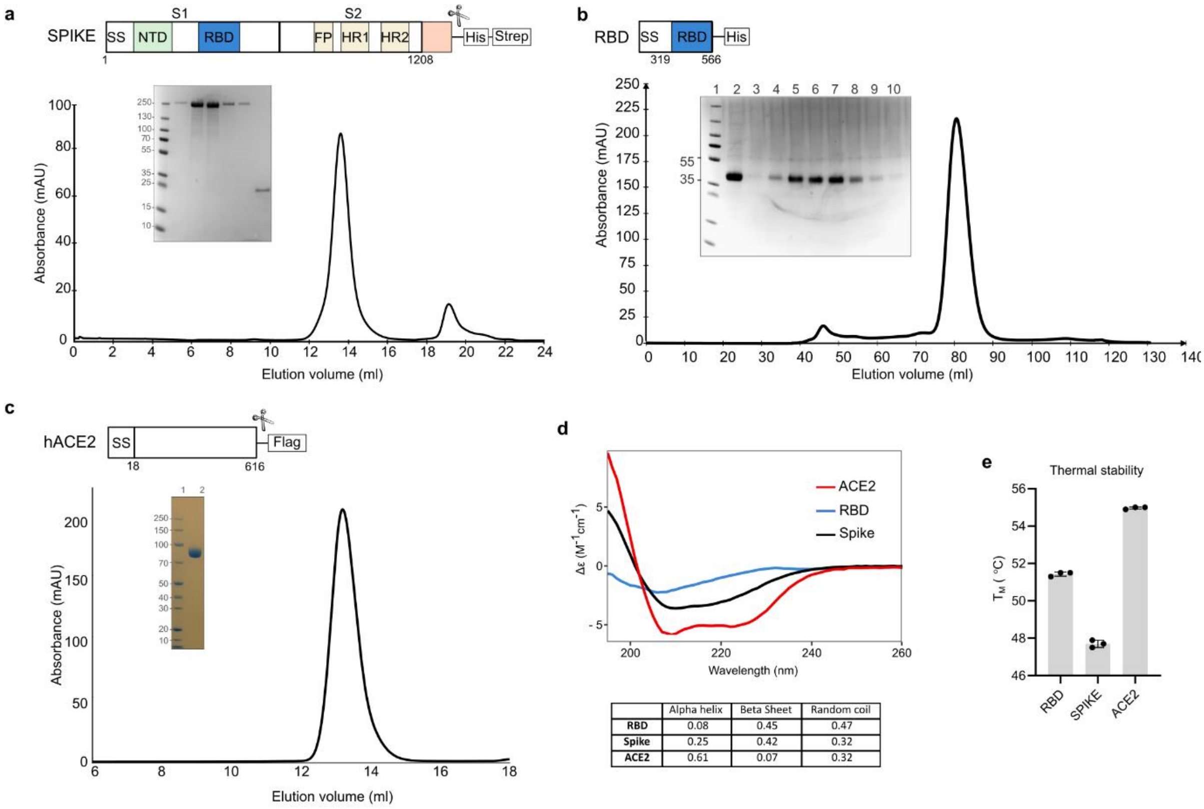

	Alpha helix	Beta Sheet	Random coil
**RBD**	0.08	0.45	0.47
**Spike**	0.25	0.42	0.32
**ACE2**	0.61	0.07	0.32

Purification and biophysical characterization of antigens.

Purified proteins showed no signs of aggregation and remained stable and monodisperse. **a** Schematic illustration of the SARS-CoV-2 spike protein. Subunit S1 comprises the N-terminal signal sequence (SS), the N-terminal domain (NTD) and the Receptor Binding Domain (RBD), while subunit S2 consists of the fusion peptide (FP) and two heptad repeats, HR1 and HR2. At the C-terminus the construct contains a T4 fibritin trimerization motif, followed by a HRV3C protease cleavage site, an 8×Histidine tag and a Twin-Strep tag. Representative gel-filtration chromatogram of the spike protein. Inset: Instant blue-stained SDS-PAGE analysis of the size exclusion chromatography run. Lane 1: protein molecular weight marker. Lanes 2 - 6: fractions eluted from the Superose 6 column. Line 7: fractions contained 3C protease. **b** The RBD comprising residues 319 - 566 was used for sybody selections. A representative gel-filtration chromatogram of RBD is shown. Inset: Instant blue-stained SDS-PAGE analysis of the size exclusion chromatography run. Lane 1: protein molecular weight marker. Line 2: pooled fractions from IMAC purification. Lanes 3 - 10: fractions eluted from the HiLoad Superdex 200 column. **c** The human receptor ACE2 comprising residues 18 - 616 was used for competition assays. A representative gel-filtration chromatogram of the ACE2 protein is

shown. Inset: Instant blue-stained SDS-PAGE analysis of the size exclusion chromatography run. Lane 1: protein molecular weight marker. Lane 2 pooled fractions eluted from the Superdex 200 column. All the three proteins (RBS, Spike and ACE2) were expressed and purified at least three times and showed identical SEC profiles. **d** Far-UV-CD spectra of the human ACE2 receptor (red), SARS-CoV-2 RBD (blue) and SARS-CoV-2 spike (black) with the corresponding secondary structure content. **e** Thermal stability analysis of RBD, spike and ACE2. Data represents the mean ± SD of three replicate experiments.

References

1. Tegunov, D. & Cramer, P. Real-time cryo – EM data pre-processing with Warp. *Nat. Methods* **16**, 1146–1152 (2020).
2. Barnes, C. O. *et al.* Structures of Human Antibodies Bound to SARS-CoV-2 Spike Reveal Common Epitopes and Recurrent Features of Antibodies. *Cell* **182**, 1–15 (2020).
