# Supplementary material for: Selection, biophysical and structural analysis of synthetic nanobodies that effectively neutralize SARS-CoV-2
Source: Nat Commun. 2020 Nov 4;11:5588. doi: 10.1038/s41467-020-19204-y (PMC7642358; doi:10.1038/s41467-020-19204-y)
Supplement: Supplementary file 3 — Description of Additional Supplementary Files [file 41467_2020_19204_MOESM3_ESM.pdf]

Description of Additional Supplementary Files

File Name: Supplementary Data 1
Description: List of sybody sequences
