# Supplementary material for: Selection, biophysical and structural analysis of synthetic nanobodies that effectively neutralize SARS-CoV-2
Source: Nat Commun. 2020 Nov 4;11:5588. doi: 10.1038/s41467-020-19204-y (PMC7642358; doi:10.1038/s41467-020-19204-y)
Supplement: Supplementary file 4 — Supplementary Data 1 [file 41467_2020_19204_MOESM4_ESM.pdf]

Sybody	Sequence
1	QVQLVESGGGLVQAGGSLRLSCAASGFPVNTSEMAWYRQAPGKEREWVAAIESSGDETYYADSVKGRFTISRD NAKNTVYLQMNSLKPEDTAVYYCYVYVGGSYLGQGTQVTVS
2	QVQLVESGGGLVQAGGSLRLSCAASGFPVSNEEMTWYRQAPGKEREWVAAIASNGNQTEYADSVKGRFTISR DNAKNTVYLQMNSLKPEDTAVYYCYVYVGASYIGQGTQVTVS
3	QVQLVESGGGLVQAGGSLRLSCAASGFPVSHTEMTWYRQAPGKEREWVAAIESQGVYTHYADSVKGRFTISRD NAKNTVYLQMNSLKPDDTAVYYCYVYVGGGYIGQGTQVTVS
4	QVQLVESGGGLVQAGGSLRLSCAASGFPVNGFNMEWYRQAPGKEREWVAAITSEGNYTYYADSVKGRFTISRD NAKNTVYLQMNSLKPEDTAVYYCYVYVGQSYIGQGTQVTVS
5	QVQLVESGGGLVQAGGSLRLSCAASGFPVAQQEMTWYRQAPGKEREWVAAISSIGSITHYADSVKGRFTISRD NAKNTVYLQMNSLKPEDTAVYYCYVYVGASYIGQGTQVTVS
6	QVQLVESGGGLVQAGGSLRLSCAASGFPVNHTRMYWYRQAPGKEREWVAAIQSHGQNTFYADSVKGRFTISR DNAKNTVYLQMNSLKPEDTAVYYCYVWVGNQYWGQGTQVTVS
7	QVQLVESGGGLVQAGGSLRLSCAASGFPVNAEEMEWYRQAPGKEREWVAAISSSGDWTYYADSVKGRFTISR DNAKNTVYLQMNSLKPEDTAVYYCLVYVGSTYIGQGTQVTVS
8	QVQLVESGGGLVQAGGSLRLSCAASGFPVKSYEMEWYRQAPGKEREWVAAISSYGEYTEYADSVKGRFTISRDN AKNTVYLQMNSLKPEDTAVYYCYVWVGDSYLGQGTQVTVS
9	QVQLVESGGGLVQAGGSLRLSCAASGFPVGQQHMYWYRQAPGKEREWVAAIYSYGHITKYADSVKGRFTISRD NAKNTVYLQMNSLKPEDTAVYYCWVYVGDYYEGQGTQVTVS
10	QVQLVESGGGLVQAGGSLRLSCAASGFPVESAEMEWYRQAPGKEREWVAAITSQGTITEYADSVKGRFTISRDN AKNTVYLQMNSLKPEDTAVYYCYVYVGASYIGQGTQVTVS
11	QVQLVESGGGLVQAGGSLRLSCAASGFPVRTENMHWYRQAPGKEREWVAAIYSFGATMLYADSVKGRFTISRD NAKNTVYLQMNSLKPEDTAVYYCAVNVGEWYEGQGTQVTVS
12	QVQLVESGGGLVQAGGSLRLSCAASGFPVQLYWMEWYRQAPGKEREWVAAITSDGDYTEYADSVKGRFTISR DNAKNTVYLQMNSLKPEDTAVYYCYVKVGEWYYGQGTQVTVS
13	QVQLVESGGGLVQAGGSLRLSCAASGFPVENYYMRWYRQAPGKEREWVAAIESSGAETRYADSVKGRFTISRD NAKNTVYLQMNSLKPEDTAVYYCYVVVGWGYAGQGTQVTVS
14	QVQLVESGGGLVQAGGSLRLSCAASGFPVATAEMVWYRQAPGKEREWVAAIMSAGQETEYADSVKGRFTISR DNAKNTVYLQMNSLKPEDTAVYYCYVWVGKSYIGQGTQVTVS
15	QVQLVESGGGLVQAGGSLRLSCAASGFPVYEHYMRWYRQAPGKEREWVAAIQSHGNHTAYADSVKGRFTISR DNAKNTVYLQMNSLKPEDTAVYYCFVVVGNGYTGQGTQVTVS
16	QVQLVESGGGLVRAGGSLRLSCAASGFPVASQEMTWYRQAPGKEREWVAAISSSGRQTEYADSVKGRFTISRD NAKNTVYLQMNSLKPEDTAVYYCYVYVGGSYIGQGTQVTVS
17	QVQLVESGGGLVQAGGSLRLSCAASGFPVKASEMEWYRQAPGKEREWVAAIASIGYNTYYADSVKGRFTISRD NAKNTVYLQMNSLKPEDTAVYYCLVYVGATYIGQGTQVTVS

18	QVQLVESGGGLVQAGGSLRLSCAASGFPVDYKSMWWYRQAPGKEREWVAAIDSAGDTTTYADSVKGRFTISR DNAKNTVYLQMNSLKPEDTAVYYCTVWVGTTYTGQGTQVTVS
19	QVQLVESGGGLVQAGGSLRLSCAASGFPVYNEEMTWYRQAPGKEREWVAAIASYGRLTEYADSVKGRFTISRD NAKNTVYLQMNSLKPEDTAVYYCYVYVGQSYIGQGTQVTVS
21	QVQLVESGGGLVQAGGSLRLSCAASGFPVKESEMTWYRQARGKEREWVAAINSHGMTTHYADSVKGRFTISR DNAKNTVYLQMNSLKPEDTAVYYCYVYVGGSYIGQGTQVTVS
22	QVQLVESGGGLVQAGGSLRLSCAASGFPVNHYEMEWYRQAPGREREWVAAIMDSTGYETAYADSVKGRFTIS RDNAKNTVYLQMNSLKPEDTAVYYCYVYVGASYIGQGTQVTVS
23	QVQLVESGGGLVQAGGSLRLSCAASGFPVESENMHWYRQAPGKEREWVAAIYSTGGWTLYADSVKGRFTISR DNAKNTVYLQMNSLKPEDTAVYYCAVQVGYWYEGQGTQVTVS
24	QVQLVESGGGLVQAGGSLRLSCAASGFPVSTAEMEWYRQAPGKEREWVAAISSSGTWTYYADSVKGRFTISRD NAKNTVYLQMNSLKPEDTAVYYCYVWVGSSYLGQGTQVTVS
25	QVQLVESGGGLVQAGGSLRLSCAASGFPVESTEMTWYRQAPGKEREWVAAIESEGHGTEYADSVKGRFTISRD NAKNTVYLQMNSLKPEDTAVYYCYVYVGAGYIGQGTQVTVS
26	QVQLVESGGGLVQAGGSLRLSCAASGFPVGQENMHWYRQAPGKEREWVAAIYSYGNFTLYADSVKGRFTISRD NAKNTVYLQMNSLKPEDTAVYYCEVXVGVWYAGLGTQVTVS
27	QVQLVESGGGLVQAGGSLRLSCAASGFPVKASEMVWYRQAPGKEREWVAAILSQGHATEYADSVKGRFTISRD NAKNTVYLQMNSLKPEDTAVYYCYVWVGRSYIGQGTQVTVS
28	QVQLVESGGGLVQAGGSLRLSCAASGFPVYSAEMEWYRQAPGKEREWVAAISSYGTNTYYADSVKGRFTISRD NAKNTVYLQMNSLKPEDTAVYYCYVYVGSSYIGQGTQVTVS
29	QVQLVESGGGLVQAGGSLRLSCAASGFPVHYTYMRWYRQAPGKEREWVAAIKSYGQHTTYADSVKGRFTISRD NAKNTVYLQMNSLKPEDTAVYYCEVVGETYRGQGTQVTVS
30	QVQLVESGGGLVQAGGSLRLSCAASGFPVWYKEMEWYRQAPGKEREWVAAITSAGHHTYYADSVKGRFTISR DNAKNTVYLQMNSLKPEDTAVYYCYVYVGESYIGQGTQVTVS
31	QVQLVESGGGLVQAGGSLRLSCAASGFPVWSAEMTWYRQAPGKEREWVAAINSHGRITEYADSVKGRFTISRD NAKNTVYLQMNGLKPEDTAVYYCYVYVGKSYIGQGTQVTVS
32	QVQLVESGGGLVQAGGSLRLSCAASGFPVAHKSMWWYRQAPGKEREWVAAIESTGDTTRYADSVKGRFTISR DNAKNTVYLQMNSLKPEDTAVYYCVVWVGEVYRGQGTQVTVS
33	QVQLVESGGGLVQAGGSLRLSCAASGFPVKTAEMEWYRQAPGKEREWVAAISSTGKSTFYADSVKGRFTISRDN AKNTVYLQMNSLKPEDTAVYYCHVYVGRTYIGQGTQVTVS
34	QVQLVESGGGLVQAGGSLRLSCAASGFPVAYYEMEWYRQAPGKEREWVAAITSSGVSTYYADSVKGRFTISRD NAKNTVYLQMNSLKPEDTAVYYCYVFVGXXYIGQGTQVTVS
35	QVQLVESGGGLVQAGGSLRLSCAASGFPVNYTIMFWYRQAPGKEREWVAAIKSTGEHTLYADSVKGRFTISRDN AKNTVYLQMNSLKPEDTAVYYCNVKDAGQGWQEYDYWGQGTQVTVS
36	QVQLVESGGGLVQAGGSLRLSCAASGFPVEWKHMTWYRQAPGKEREWVAAIASTGAYTHYADSVKGRFTISR DNAKNTVYLQMNSLKPEDTAVYYCNVKDWGSKTTYYDYWGQGTQVTVS

37	QVQLVESGGGLVQAGGSLRLSCAASGFPVYNTWMEWYRQAPGKEREWVAAITSYGFHTYYADSVKGRFTISRDNAKNTVYLQMNSLKPEDTAVYYCNVKDEGNTTAYYDYWGQGTQVTVS
38	QVQLVESGGGLVQAGGSLRLSCAASGFPVYWAHMTWYRQAPGKEREWVAAIVSSGAYTAYADSVKGRFTISRDNAKNTVYLQMNSLKPEDTAVYYCNVKDFGTQEHYYDYWGQGTQVTVS
39	QVQLVESGGGPVQAGGSLRLSCAASGFPVMWSHMHWYRQAPGKEREWVAAIVSYGAYTIYADSVKGRFTISRDNAKNTVYLQMNSLKPEDTAVYYCNVKDFGGYRYYYDYWGQGTQVTVS
40	QVQLVESGGGLVQAGGSLRLSCAASGFPVQGTWMEWYRQAPGKEREWVAAITSVGYRTYYADSVKGRFTISRDNAKNTVYLQMNSLKPEDTAVYYCNVKDEGAIAKNYDYWGQGTQVTVS
41	QVQLVESGGGLVQAGGSLRLSCAASGFPVEHQHMHWYRQAPGKEREWVAAIVSTGHYTIYADSVKGRFTISRDNAKNTVYLQMNSLKPEDTAVYYCNVKDWGDTSQYYDYWGQGTQVTVS
42	QVQLVESGGGLVQAGGSLRLSCAASGFPVYNTWMEWYRQAPGKEREWVAAITSWGFKTYYADSVKGRFTISRDNAKNTVYLQMNSLKPEDTAVYYCNVKDEGYTGYYYDYWGQGTQVTVS
43	QVQLVESGGGLVQAGGSLRLSCAASGFPVEWTHMHWYRQAPGKEREWVAAIASSGAYTVYADSVKGRFTISRDNAKNTVYLQMNSLKPEDTAVYYCNVKDWGSQDRYYDYWGQGTQVTVS
44	QVQLVESGGGLVQAGGSLRLSCAASGFPVERKHMLWYRQSPGKEREWVAAIVSYGNYTTYADSVKGRFTISRDNAKNTVYLQMNSLKPEDTAVYYCNVKDHGFNTVLYDYWGQGTQVTVS
45	QVQLVESGGGLVQAGGSLRLSCAASGFPVAGTWMEWYRQAPGKEREWVAAITSYGYRTYYADSVKGRFTISRDNAKNTVYLQMNSLKPEDTAVYYCNVKDEGKSSQVYDYWGQGTQVTVS
46	QVQLVESGGGLVQAGGSLRLSCAASGFPVNYTIMFWYRKAPGKEREWVAAIKSHGATTLYADSVKGRFTISRDNAKNTVYLQMNSLKPEDTAVYYCNVKDVGNDQKSYDYWGQGTQVTVS
47	QVQLVESGGGLVQAGGSLRLSCAASGFPVVWAHMHWYRQAPGKEREWVAAITSEGAHTIYADSVKGRFTISRDNAKNTVYLQMNSLKPEDTAVYYCNVKDWGTYSTYYDYWGQGTQVTVS
48	QVQLVESGGGLVQAGGSLRLSCAASGFPVAHWWMEWYRQAPGKEREWVAAIFSEGNWTHYADSVKGRFTISRDNAKNTVYLQMNSLKPEDTAVYYCNVKDTGATQFAYDYWGQGTQVTVS
49	QVQLVESGGGLVQAGGSLRLSCAASGFPVHYTHMHWYRQAPGKEREWVAAIASSGAYTVYADSVKGRFTISRDNAKNTVYLQMNSLKPEDTAVYYCNVKDWGTYNTYYDYWGQGTQVTVS
50	QVQLVESGGGLVQAGGSLRLSCAASGFPVQYEHMHWYRQAPGKEREWVAAIVSEGAYTHYADSVKGRFTISRDNAKNTVYLQMNSLKPEDTAVYYCNVKDWGWLAQYYDYWGQGTQVTVS
51	QVQLVESGGGLVQAGGSLRLSCAASGFPVKETWMHWYRQAPGKEREWVAAITSFGYKTYYADSVKGRFTISRDNAKNTVYLQMNSLKPEDTAVYYCNVKDAGDVTHQYDYWGQGTQVTVS
52	QVQLVESGGGLVQAGGSLRLSCAASGFPVNYNWMEWYRQAPGKEREWVAAITSWGYKTYYADSVKGRFTISRDNAKNTVYLQMNSLKPEDTAVYYCNVKDEGMWQHYYDYWGQGTQVTVS
53	QVQLVESGGGLVQAGGSLRLSCAASGFPVYATVMHWYRQAPGKEREWVAAIYSTGMWTLYADSVKGRFTISRDNAKNTVYLQMNSLKPEDTAVYYCNVKDEGYDYYYYDYWGQGTQVTVS
54	QVQLVESGGGLVQAGGSLRLSCAASGFPVWNTWMEWYRQAPGKEREWVAAITSYGFKTYYADSVKGRFTISRDNAKNTVYLQMNSLKPEDTAVYYCNVKDEGNSQSHYDYWGQGTQVTVS

56	QVQLVESGGGLVQAGGSLRLSCAASGFPVEMWSMEWYRQAPGKEREWVAAIMSFGYQTWYADSVKGRFTISRDNAKNTVYLQMNSLKPEDTAVYYCNVKDAGNSKALYDYWGQGTQVTVS
57	QVQLVESGGGLVQAGGSLRLSCAASGFPVEHDHMHWYRQAPGKEREWVAAIVSQGAYTVYADSVKGRFTISRDNAKNTVYLQMNSLKPEDTAVYYCNVKDWGRAGARYDYWGQGTQVTVS
58	QVQLVESGGGLVQAGGSLRLSCAASGFPVDAAWMEWYRQAPGKEREWVAAITSYGYRTYYADSVKGRFTISRDNAKNTVYLQMNSLKPEDTAVYYCNVKDMDRWRTTYDYWGQGTQVTVS
60	QVQLVESGGGLVQAGGSLRLSCAASGFPVVAWQMTWYRQAPGKEREWVAAIRSFGVSTHYADSVKGRFTISRDNAKNTVYLQMNSLKPEDTAVYYCNVKDWGYEYEGYDYWGQGTQVTVS
61	QVQLVESGGGLVQAGGSLRLSCAASGFPVQHEWMEWYRQAPGKEREWVAAITSYGYRTYYADSVKGRFTISRDNAKNTVYLQMNSLKPEDTAVYYCNVKDTGTYQAWYDYWGQGTQVTVS
62	QVQLVESGGGLVQAGGSLRLSCAASGFPVEQEHMYWYRQASGKEREWVAAIVSEGAYTAYADSVKGRFTISRDNAKNTVYLQMNSLKPEDTAVYYCNVKDWGGYQWYYDYWGQGTQVTVS
63	QVQLVESGGGLVQAGGSLRLSCAASGFPVNAEIMFWYRQAPGKEREWVAAIKSAGTTTLYADSVKGRFTISRDNAKNTVYLQMNSLKPEDTAVYYCNVKDYGAQAHYYDYWGQGTQVTVS
64	QVQLVESGGGLVQAGGSLRLSCAASGFPVYNRYMLWYRQAPGKEREWVAAITSYGYHTTYADSVKGRFTISRDNAKNTVYLQMNSLKPEDTAVYYCNVKDYGQHQQVYDYWGQGTQVTVS
66	QVQLVESGGGLVQAGGSLRLSCAASGFPEVNTWMEWYRQAPGKEREWVAAITSYGYRTYYADSVKGRFTISRDNAKNTVYLQMNSLKPEDTAVYYCNVKDEGSQSWAYDYWGQGTQVTVS
67	QVQLVESGGGLVQAGGSLRLSCAASGFPVMWAHMAWYRQAPGKEREWVAAIVSAGAYTHYADSVKGRFTISRDNAKNTVYLQMNSLKPEDTAVYYCNVKDWGTYNSYYDYWGQGTQVTVS
68	QVQLVESGGGSVQAGGSLRLSCAASGTITNIGYLGWFRQAPGKEREGVAALKTYDGYTYYADSVKGRFTVSLDNAKNTVYLQMNSLKPEDTALYYCAAARHGRDEPLSYMYYSYWGQGTQVTVS
69	QVQLVESGGGSVQAGGSLRLSCAASGYISEISYLGWFRQAPGKEREGVAALITGWGHTYYADSVKGRFTVSLDNAKNTVYLQMNSLKPEDTALYYCAAAQTGAWYPLETYIYHYWGQGTQVTVS
71	QVQLVESGGGSVQAGGSLRLSCAASGNIQHIKYLGWFRQAPGREREGVAALMTRYGQTYYADSVKGRFTVSLDNAKNTVYLQMNSLKPEDTALYYCAAAHYGDNFPLAYQAYLYWGQGTQVTVS
73	QVQLVESGGGSVQAGGSLRLSCAASGKIYQIGYLGWFRQAPGKEREGVAALMTTDGYTYYADSVKGRFTVSLDNAKNTVYLQMNSLKPEDTALYYCAAARHGRERPLHYWYYSYWGQGTQVTVS
75	QVQLVESGGGSVQAGGSLRLSCAASGYINQIYYLGWFRQAPGKEREGVAALNTYQGQTYYADSVKGRFTVSLDNAKNTVYLQMNSLKPEDTALYYCAAARWGRDEPLYHYYYSYWGQGTQVTVS
76	QVQLVESGGGLVQAGGSLRLSCAASSFPVDTYHMAWYRQAPGKEREWVAAIVSWGWRTYYADSVKGRFTISRDNAKNTVYLQMNSLKPEDTAVYYCNVKDIGAQEVHYDYWGQGTQVTVS
77	QVQLVESGGGSVQAGGSLRLSCAASGSISSITYLGWFRQAPAKSVRAVAALDTEQGETYYADSVKGRFTVSLDNAKNTVYLQMNSLKPEDTALYYCAAANQHGGYYPLDSAWYTYWGQGTQVTVS
78	QVQLVESGGGSVQAGGSLRLSCAASGYIKSIKYLGWFRQAPGKEREGVAALMTRYGETYYADSVKGRFTVSLDNAKNTVYLQMNSLKPEDTALYYCAAANYGNNWPLTGVNYWYWGQGTQVTVS

79	QVQLVESGGGSVQAGGSLRLSCAASGYIHTIEYLGWFRQAPGKEREGVAALYTLKGQTYYADSVKGRFTVSLDN<br>AKNTVYLQMNSLKPEDTALYYCAAARWGRDTPLHYQYYSYWGQGTQVTVS
81	QVQLVESGGGSVQAGGSLRLSCAASGYIHRIEYLGWFRQAPGKEREGVAALSTISGNTYYADSVKGRFTVSLDNA<br>KNTVYLQMNSLKPEDTALYYCAAARFGRAYPLHYYQYTYWGQGTQVTVS
82	QVQLVESGGGSVQAGGSLRLSCAASGTINSIHYLGWFRQAPGKEREGVAALDTYIGYTYYADSVKGRFTVSLDNA<br>KNTVYLQMNSLKPEDTALYYCAAARWGRTSPLHWYYYTYWGQGTQVTVS
83	QVQLVESGGGSVQAGGSLRLSCAASGGITHIVYLGWFRQAPGKEREGVAALMTRWGTTYYADSVKGRFTVSLD<br>NAKNTVYLQMNSLKPEDTALYYCAAAKYGQNFPLSYHAYRYWGQGTQVTVS
84	QVQLVESGGGSVQAGGSLRLSCAASGYIKHIEYLGWFRQAPGKEREGVAALKTSSGSTYYADSVKGRFTVSLDNA<br>KNTVYLQMNSLKPEDTALYYCAAARYGRSDPLHYHEYSYWGQGTQVTVS
85	QVQLVESGGGSVQAGGSLRLSCAASGSISSITYLGWFRQAPGKEREGVAALVTSRGKTYYADSVKGRFTVSLDNA<br>KNTVYLQMNSLKPEDTALYYCAAASWGYTWPLYTYDYWYWGQGTQVTVS
86	QVQLVESGGGSVQAGGSLRLSCAASGTIASIIYLGWFRQAPGKEREGVAALNTWNGKTYYADSVKGRFTVSLDN<br>AKNTVYLQMNSLKPEDTALYYCAAAYSGMWYPLSNEHYGYWGQGTQVTVS
88	QVQLVESGGGLVQAGGSLRLSCAASGFPVWAHHMLWYRQAPGKEREWVAAIASWGANTAYADSVKGRFTIS<br>RDNAKNTVYLQMNSLKPEDTAVYYCNVKDSGQYRENYDYWGQGTQVTVS
90	QVQLVESGGGSVQAGGSLRLSCAASGAINQIYYLGWFRQAPGKEREGVAALSTKYGETYYADSVKGRFTVSLDN<br>AKNTVYLQMNSLKPEDTALYYCAAARWGRQYPLTFVYYSYWGQGTQVTVS
93	QVQLVESGGGSVQAGGSLRLSCAASGHIAQIEYLGWFRQAPGKEREGVAALSTNQGYTYYADSVKGRFTVSLDN<br>AKNTVYLQMNSLKPEDTALYYCAAARWGRTYPLSYMAYTYWGQGTQVTVS
94	QVQLVESGGGSVQAGGSLRLSCAASGYITMIEYLGWFRQAPGKEREGVAALNTHTGGTYYADSVKGRFTVSLD<br>NAKNTVYLQMNSLKPEDTALYYCAAARWGRYEPLHYAYYSYWGQGTQVTVS
95	QVQLVESGGGSVQAGGSLRLSCAASGNIYNIKYLGWFRQAPGKEREGVAALMTRYGETYYADSVKGRFTVSLDN<br>AKNTVYLQMNSLKPEDTALYYCAAASYGANWPLVSAAYTYWGQGTQVTVS
97	QVQLVESGGGSVQAGGSLRLSCAASGAISTIEYLGWFRQAPGREREGVAALYTERGYTYYADSVKGRFTVSLDNA<br>KNTVYLQMNSLKPEDTALYYCAAARYGHAQAPLHYFWYGYWGQGTQVTVS
100	QVQLVESGGGSVQAGGSLRLSCAASGSISSITYLGWFRQAPGKEREGVAALVTSDGRTYYADSVKGRFTVSLDNA<br>KNTVYLQMNSLKPEDTALYYCAAANWGYSWPLYQTEYWYWGQGTQVTVS
