# Supplementary material for: Selection, biophysical and structural analysis of synthetic nanobodies that effectively neutralize SARS-CoV-2
Source: Nat Commun. 2020 Nov 4;11:5588. doi: 10.1038/s41467-020-19204-y (PMC7642358; doi:10.1038/s41467-020-19204-y)
Supplement: Supplementary file 5 — Reporting Summary [file 41467_2020_19204_MOESM5_ESM.pdf]

nature research

Corresponding author(s): Christian Loew

Last updated by author(s): Sep 19, 2020

Reporting Summary

Nature Research wishes to improve the reproducibility of the work that we publish. This form provides structure for consistency and transparency in reporting. For further information on Nature Research policies, see our Editorial Policies and the Editorial Policy Checklist.

Statistics

For all statistical analyses, confirm that the following items are present in the figure legend, table legend, main text, or Methods section.

n/a	Confirmed	
☐	☒	The exact sample size (*n*) for each experimental group/condition, given as a discrete number and unit of measurement
☐	☒	A statement on whether measurements were taken from distinct samples or whether the same sample was measured repeatedly
☒	☐	The statistical test(s) used AND whether they are one- or two-sided<br>*Only common tests should be described solely by name; describe more complex techniques in the Methods section.*
☒	☐	A description of all covariates tested
☒	☐	A description of any assumptions or corrections, such as tests of normality and adjustment for multiple comparisons
☐	☒	A full description of the statistical parameters including central tendency (e.g. means) or other basic estimates (e.g. regression coefficient) AND variation (e.g. standard deviation) or associated estimates of uncertainty (e.g. confidence intervals)
☒	☐	For null hypothesis testing, the test statistic (e.g. *F*, *t*, *r*) with confidence intervals, effect sizes, degrees of freedom and *P* value noted<br>*Give P values as exact values whenever suitable.*
☒	☐	For Bayesian analysis, information on the choice of priors and Markov chain Monte Carlo settings
☒	☐	For hierarchical and complex designs, identification of the appropriate level for tests and full reporting of outcomes
☒	☐	Estimates of effect sizes (e.g. Cohen's *d*, Pearson's *r*), indicating how they were calculated

Our web collection on statistics for biologists contains articles on many of the points above.

Software and code

Policy information about availability of computer code

Data collection	not applicable
Data analysis	Interferometry data were processed with the Octet Data Analysis software v10.0 (ForteBio). The MST data were analyzed using the MO. Affinity Analysis software (NanoTemper Technologies). The neutralization data were fitted using Prism 8 (GraphPad).

For manuscripts utilizing custom algorithms or software that are central to the research but not yet described in published literature, software must be made available to editors and reviewers. We strongly encourage code deposition in a community repository (e.g. GitHub). See the Nature Research guidelines for submitting code & software for further information.

Data

Policy information about availability of data

All manuscripts must include a data availability statement. This statement should provide the following information, where applicable:

- Accession codes, unique identifiers, or web links for publicly available datasets
- A list of figures that have associated raw data
- A description of any restrictions on data availability

The scattering data and models of Sy23, RBD and their complex are deposited into SASBDB (www.sasbdb.org), entries SASDJF4, SASDJG4 and SASDJH4 respectively. The cryo-EM density maps of SARS-CoV-2 spike glycoprotein with Sb23 bound were deposited in the Electron Microscopy Data Bank (EMDB) with accession codes EMD-11616 (1-up) and EMD-11617 (2-up). The corresponding models were deposited in the Protein Data Bank (PDB) with accession codes 7A25 (1-up) and 7A29 (2-up). The sequences of all the selected sybodies from this study are provided in Supplementary Data 1. The MST, BLI and neutralization data are available under https://github.com/tania-custodio/Sb23 and in the Source File. Any other experimental data that support the findings of this study are available from the corresponding authors upon request.

Field-specific reporting

Please select the one below that is the best fit for your research. If you are not sure, read the appropriate sections before making your selection.

☒ Life sciences ☐ Behavioural & social sciences ☐ Ecological, evolutionary & environmental sciences

For a reference copy of the document with all sections, see nature.com/documents/nr-reporting-summary-flat.pdf

Life sciences study design

All studies must disclose on these points even when the disclosure is negative.

Sample size	Sample size is not relevant for this study, no calculations were made.
Data exclusions	No data was excluded from any analysis
Replication	Replicate experiments are indicated in the respective legends
Randomization	This study reports the biophysical and structural characterization of protein complex, and randomization is not relevant.
Blinding	This study reports the biophysical and structural characterization of protein complex, and blinding is not relevant.

Reporting for specific materials, systems and methods

We require information from authors about some types of materials, experimental systems and methods used in many studies. Here, indicate whether each material, system or method listed is relevant to your study. If you are not sure if a list item applies to your research, read the appropriate section before selecting a response.

Materials & experimental systems

n/a	Involved in the study	
☐	☒	Antibodies
☐	☒	Eukaryotic cell lines
☒	☐	Palaeontology and archaeology
☒	☐	Animals and other organisms
☒	☐	Human research participants
☒	☐	Clinical data
☒	☐	Dual use research of concern

Methods

n/a	Involved in the study	
☒	☐	ChIP-seq
☒	☐	Flow cytometry
☒	☐	MRI-based neuroimaging

Antibodies

Antibodies used	Mouse monoclonal anti myc, Sigma #M4439 ELISA. Dilution of 1:2000
Validation	The antibody was validated by ELISA

Eukaryotic cell lines

Policy information about cell lines

Cell line source(s)	FreeStyle ™ 293-F purchase from Invitrogen. This cells are derived from HEK293-F cells.
Authentication	No authentication of the FreeStyle ™ 293-F cell line was done.
Mycoplasma contamination	We confirmed that all cell lines tested negative for mycoplasma contamination.
Commonly misidentified lines (See ICLAC register)	No commonly misidentified cell lines were used.
